# Rosemary Essential Oil-Loaded Lipid Nanoparticles: In Vivo Topical Activity from Gel Vehicles

**DOI:** 10.3390/pharmaceutics9040048

**Published:** 2017-10-21

**Authors:** Lucia Montenegro, Lorella Pasquinucci, Agata Zappalà, Santina Chiechio, Rita Turnaturi, Carmela Parenti

**Affiliations:** 1 Department of Drug Sciences, Pharmaceutical Technology Section, University of Catania, Viale A. Doria 6, 95125 Catania, Italy; 2 Department of Drug Sciences, Medicinal Chemistry Section, University of Catania, Viale A. Doria 6, 95125 Catania, Italy; lpasquin@unict.it (L.P.); rita.turnaturi@tiscali.it (R.T.); 3 Department of Biomedical and Biotechnological Sciences, Physiology Section, University of Catania, Via Santa Sofia, 87, 95125 Catania, Italy; azappala@unict.it; 4 Department of Drug Sciences, Pharmacology and Toxicology Section, University of Catania, Viale A. Doria 6, 95125 Catania, Italy; santinachiechio@unict.it (S.C.); cparenti@unict.it (C.P.)

**Keywords:** rosemary essential oil, lipid nanoparticles, skin hydration, skin elasticity, in vivo evaluation, essential oils

## Abstract

Although rosemary essential oil (EO) shows many biological activities, its topical benefits have not been clearly demonstrated. In this work, we assessed the effects on skin hydration and elasticity of rosemary EO after topical application via gel vehicles in human volunteers. To improve its topical efficacy, rosemary EO was loaded into lipid nanoparticles (NLCs) consisting of cetyl palmitate as a solid lipid, and non-ionic surfactants. Such NLCs were prepared using different ratios of EO/solid lipid and those containing EO 3% *w*/*w* and cetyl pamitate 7% *w*/*w* were selected for in vivo studies, showing the best technological properties (small particle size, low polydispersity index and good stability). Gels containing free EO or EO-loaded NLCs were applied on the hand skin surface of ten healthy volunteers twice a day for one week. Skin hydration and elasticity changes were recorded using the instrument Soft Plus. Gels containing EO-loaded NLCs showed a significant increase in skin hydration in comparison with gels containing free EO. Skin elasticity increased, as well, although to a lesser extent. The results of this study point out the usefulness of rosemary EO-loaded NLCs for the treatment of cutaneous alterations involving loss of skin hydration and elasticity.

## 1. Introduction

In recent years, the increasing demand for pharmaceutical and cosmetic products based on natural active ingredients has brought researchers to focus their attention on essential oils (EOs). Traditionally, EOs have been used as fragrances in food and cosmetics but, in the last decades, many investigations have highlighted their numerous biological and medicinal properties, demonstrating their antioxidant, preservative, antimicrobial, antiviral, fungicidal, insecticidal, anti-inflammatory, analgesic, sedative and anticancer activities [1,2,3,4,5,6,7].

EOs are volatile, lipophilic liquid or semiliquid, complex mixtures of low molecular weight organic compounds produced by plants as secondary metabolites whose main components consist of monoterpenoids and sesquiterpenoids, phenylpropanoids and short-chained aliphatics. Due to the chemical structures of their constituents, EOs can be easily degraded after exposure to humidity, heat, oxygen and light, owing to chemical and enzymatic reactions (oxidation, isomerization, cyclization, and dehydrogenation), which may occur during the manufacturing and handling processes [8]. The presence of degradation products in EOs could impair their biological activity. For example, topical application of EOs containing oxidized terpenoids may show skin-sensitizing effects, thus inducing allergic contact dermatitis [9,10].

To overcome the drawbacks of EOs, such as high volatility, easy degradation, high lipophilicity and poor membrane permeability, several researchers have suggested the encapsulation of these active ingredients into nanocarriers [11].

Advantages, including the ability to incorporate lipophilic and hydrophilic drugs, controlled drug release, drug targeting, increased drug stability and bioavailability, safety, easy scale-up and low cost of production, have boosted the use of solid lipid nanoparticles (SLNs) and nanostructured lipid carriers (NLCs) as nanocarriers for different administration routes [12,13,14,15,16,17]. Recent investigations have revealed the usefulness of SLNs as carriers for EO delivery. EOs from the genera *Boswellia* and *Commiphora*, exhibiting antimicrobial, anti-inflammatory, and antitumor activities, were efficiently loaded into SLNs designed for oral delivery, providing increased anticancer activity compared to free EOs [18]. Moghimipour et al. [19] prepared SLNs encapsulating the essential oil extracted from the leaves of *Zataria multiflora* and concluded that SLNs are suitable carriers for essential oils. The same conclusion was reported in a study assessing SLNs loaded with *Nigella sativa* essential oil [20]. Two different SLN formulations were investigated in vitro as carriers for the topical and transdermal delivery of *Artemisia arborescens* essential oil [21]. This study pointed out the high physical stability of these formulations, along with their ability to target the essential oil to the skin layers, while retaining in vitro antiviral activity.

Rosemary (*Rosmarinus officinalis* L.) essential oil has been attributed antioxidant, anti-inflammatory, antimicrobial, fungicidal, and anticancer activity, mainly owing to its flavonoids and terpenes content [22,23,24,25]. Investigations on rosemary aqueous extracts pointed out the antioxidant activity of this extract and its ability to control lipoperoxidation of skin lipids. Therefore, rosemary aqueous extracts have been suggested as a useful tool for the prevention and treatment of skin disorders, including age-related skin damages [26]. However, to date, topical benefits of rosemary essential oil have not been clearly demonstrated.

In this work, we evaluated the effects on skin hydration and elasticity of rosemary essential oil after topical application via gel vehicles in human volunteers. To improve its topical efficacy, rosemary essential oil was loaded into lipid nanoparticles, whose safety has been previously reported [27].

After a one-week topical treatment with gels containing two different percentages of rosemary EO-loaded lipid nanoparticles, a significant increase in skin hydration was observed in comparison with gels containing the same percentages of free rosemary EO. Skin elasticity increased as well, although to a lesser extent. These results suggest that the design of suitable lipid nanocarriers could be a successful approach to improve rosemary EO topical benefits.

## 2. Materials and Methods

### 2.1. Materials

Polyoxyethylene-20-oleyl ether (Brij 98^®^, Oleth-20) was bought from Sigma-Aldrich (Milan, Italy). Methylchloroisothiazolinone and methylisothiazolinone (Kathon CG^®^), and Imidazolidinyl urea (Gram 1^®^) were kindly supplied by Sinerga (Milan, Italy). Carbopol Ultrez 21^®^ (Carbopol), Cetyl Palmitate (CP), Glyceryl Oleate (GO) and Triethanolamine (TEA) were bought from ACEF (Fiorenzuola D'Arda, Italy). Solubilisant Gamma 2428^®^ (octoxynol-11, PEG-40 hydrogenated castor oil, polysorbate 20; Solubilisant) was a kind gift of Gattefossé Italia (Milan, Italy). Rosemary oil (Rosmarinus officinalis flower oil, EO) was a kind gift of Exentiae (Catania, Italy). According to the data sheet provided by the manufacturer, rosemary oil contained 1,8-cineol (25–60%), alpha-pinene (8–20%), beta-pinene (2–10%), beta-caryophyllene (4–10%), camphor (7–8%), camphene (3–8%), limonene (2–3%), myrcene (1–2%), terpinolene (0.2–0.5%), 3-carene (0.15–0.5%).

### 2.2. Lipid Nanoparticles Preparation

Unloaded SLNs and EO-loaded NLCs, whose composition is reported in Table 1, were prepared using the phase inversion temperature (PIT) method [27]. The aqueous phase (double distilled water containing 0.35% (*w*/*w*) imidazolidinyl urea and 0.05% (*w*/*w*) methylchloroisothiazolinone and methylisothiazolinone as preservatives) and the oil phase (cetyl palmitate, emulsifier and co-emulsifier, different percentages *w*/*w* of EO) were separately heated at 85 °C. Two series of NLCs were prepared: NLCs BEO1–4 were obtained using a constant amount of lipid phase (7% *w*/*w*) with different ratios solid lipid/liquid lipid, while NLCs CEO1–3 contained a constant amount of solid lipid (7% *w*/*w*) and different percentages of liquid lipid.

When both phases were at the same temperature, the water phase was slowly added to the oil phase while being stirred and then the resulting colloidal system was cooled to room temperature, under constant agitation. The turbid mixture turned into a clear system at the phase inversion temperature (PIT), whose value was determined using a conductivity meter, model 525 (Crison, Modena, Italy).

### 2.3. Nanoparticles Characterization

#### 2.3.1. Transmission Electron Microscopy

For negative-staining electron microscopy, 5 µL of lipid nanocarriers dispersions were placed on a 200-mesh formvar copper grid (TAAB Laboratories Equipment, Berks, UK) and allowed to be adsorbed. Then the surplus was removed by filter paper. A drop of 2% (*w*/*v*) aqueous solution of uranyl acetate was added over 2 min. After removing the surplus, the sample was dried at room temperature before imaging it with a transmission electron microscope (model JEM 2010, Jeol, Peabody, MA, USA) operating at an acceleration voltage of 200 kV.

#### 2.3.2. Size and Zeta Potential Measurements

Mean particle size and size distribution were determined in double distilled water by a dynamic light scattering (DLS) using a Zetasizer NanoZS (ZEN 3600, Malvern, Germany).

All the samples were diluted at 1:5 (sample/water) and thermostated at 25 °C for 2 min prior to the measurement. The instrument performed particle sizing by means of a 4 mW laser diode operating at 670 nm. Using the same instrument, ζ potential was determined by the technique of laser Doppler velocimetry, after diluting the sample with KCl 1 mM (pH 7.0), according to a procedure already reported [28].

Each measurement was performed in triplicate and values were expressed as mean ± standard deviation (S.D.).

#### 2.3.3. Stability Tests

Samples of lipid nanoparticles were stored in airtight jars, and then kept in the dark and at room temperature for two months.

At fixed intervals (24 h, one week, two weeks, one month and two months after their preparation), the particle size and polydispersity index of the samples were measured.

### 2.4. Gel Preparation

Gel formulations were prepared using Carbopol Ultrez 21 as a gelling agent and TEA for neutralization, and their compositions are reported in Table 2. The rheological additive (0.8% *w*/*w*) was carefully dispersed in the aqueous phase, under mild agitation. The aqueous phase consisted of deionized water (gel A), deionized water containing EO and Solubilisant Gamma 2428 (gel B and B1), SLN A (gel C), NLC CEO3 (gel D and D1). As rosemary essential oil was poorly water soluble, we added a solubilizing compound to obtain aqueous gels containing different percentages of EO. All gel formulations were prepared using deionized water containing 0.35% *w*/*w* imidazolidinyl urea, 0.05% *w*/*w* methylchloroisothiazolinone and methylisothiazolinone as preservatives. The resulting mixture was left in the dark at room temperature for 24 h, to allow complete polymer hydration. Then, TEA (0.8% *w*/*w*) was added drop by drop under slow agitation, to prevent the formation of air bubbles, until the gel was formed. All formulations were stored in airtight jars, and kept in the dark and at room temperature until use.

### 2.5. In Vivo Evaluation of Gel Formulations

This experiment has been designed to assess the in vivo activity of rosemary oil-loaded lipid nanoparticles in comparison with unloaded SLNs and free rosemary oil in gel vehicles after topical application for one week. Investigations were carried out following the rules of the Declaration of Helsinki of 1975, revised in 2008. The local ethics committee declared that no approval was needed for this type of study, due to the nature of the study and the safety of the ingredients used in the formulations under investigation. Ten healthy female volunteers (average age 53 ± 4), without dermatological diseases and with normal/moderate dry skin were enrolled in the study, after giving their informed consent. Young healthy subjects were not included in this study as they showed high hydration skin levels that made them unsuitable for the evaluation of the effects of skin hydrating treatments. Freshly prepared gel samples were presented in similar containers labeled with four-digit code numbers and the study was performed in double blind. In the first part of the study, subjects were given four out of the six different gels. The volunteers were explained how to pick up and apply the samples and were instructed to apply the product (about 2 mg) onto two different areas over the back of their hands, twice a day (morning and evening) for a period of one week.

Measurements of skin hydration and elasticity were performed at baseline (prior to the treatment) and after one week of treatment. Measurements were taken under controlled temperature (22 ± 1 °C) and humidity (35 ± 5%) conditions, after 30 min of acclimatization. The volunteers were requested not to apply the gels the morning before the final measurement. The use of other products was not allowed throughout the study, apart from normal hygiene treatment. Skin analyses were performed using specific probes of the instrument Soft Plus (Callegari Srl, Parma, Italy). These probes determined skin hydration by capacity measurements in the range 0–100 u.c. (arbitrary units) with a resolution of 1 u.c. and 5% precision, while stress/deformation of skin by suction application allowed measurements of skin elasticity in the range 0–50 u.c. with a resolution of 1 u.c. and 10% precision. Each measurement was performed in triplicate.

In the second part of the study, after a one-month wash-out, the same subjects were requested to apply the remaining two gels and the test was performed in the same conditions and using the same procedures and instrumental analyses described above.

Statistical analysis of the results was performed using Students’ *t* test (*p* < 0.05).

## 3. Results and Discussion

### 3.1. Lipid Nanoparticles Characterization

As reported in the literature [29], the use of solid lipids to form the lipid nanocarrier core leads to nanoparticles defined as SLNs while the use of mixtures of solid and liquid lipids results in NLCs. Therefore, in this work, lipid nanoparticles loading rosemary EO were regarded as NLCs, being that their lipid phase was a mixture of solid lipid (cetyl palmitate) and liquid lipid (rosemary EO).

As shown in Table 3, unloaded lipid nanoparticles containing only the solid lipid cetyl palmitate (SLN A) showed small sizes (36.45 nm) and narrow dimensional distribution (polydispersity index 0.208), in accordance with previous studies [27,30]. When rosemary oil was mixed in different ratios with cetyl palmitate, NLCs with different mean sizes and polydispersity indexes were obtained. When the total amount of lipid phase (solid lipid plus oil) was maintained constant (7% *w*/*w*, NLC BEO1–4), an increment of oil content led to an increase of mean particle size. For these lipid nanoparticles, polydispersity index values were higher than 0.300, thus indicating that the distribution did not consist of a single size mode.

**Figure 1:**
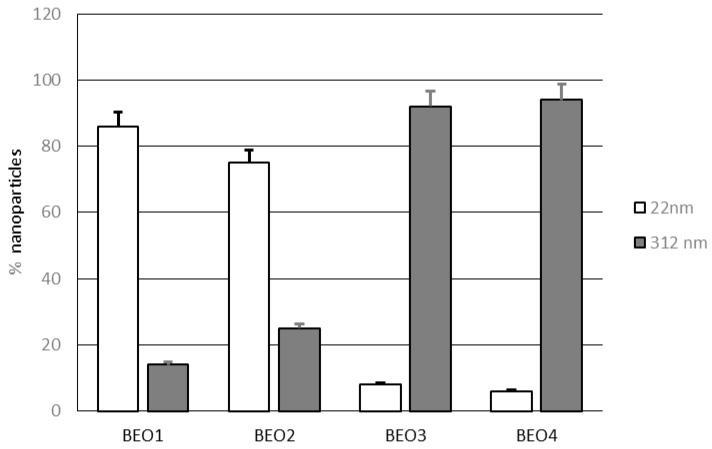
Percentages of nanoparticles with different mean size (22 and 312 nm) in lipid nanoparticles BEO1–4.

As illustrated in Figure 1, two different populations of lipid nanoparticles were observed in NLCs BEO1–4, one with small sizes (mean size 22 nm) and the other with bigger sizes (mean size 312 nm). At a lower rosemary EO content (1–2% *w*/*w*), the percentage of small sized nanoparticles was higher than that of large nanoparticles. When the amount of essential oil used to prepare these nanocarriers was raised to 4% *w*/*w*, the percentage of large nanoparticles became preponderant.

**Figure 2:**
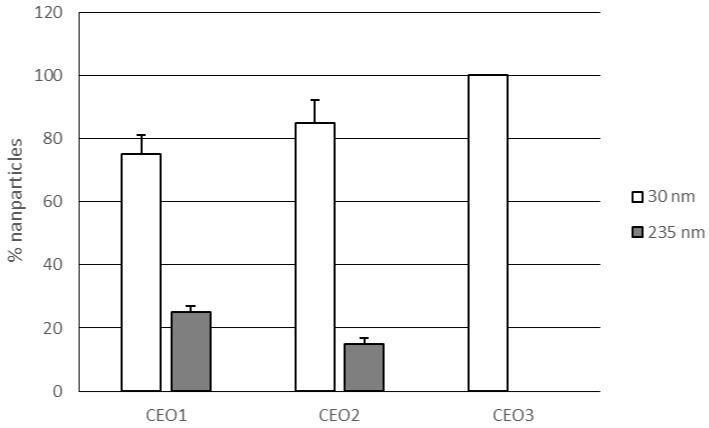
Percentages of nanoparticles with different mean size (30 and 235 nm) in lipid nanoparticles CEO1–3.

The opposite trend was observed for lipid nanoparticles obtained using a constant amount of solid lipid (7% *w*/*w*) while increasing the content of rosemary EO (1–3% *w*/*w*). These NLCs (CEO1–3) showed a decrease of particle sizes and polydispersity index values as the percentage of rosemary EO increased (see Table 3). NLC CEO1 and NLC CEO2, loading 1–2% of rosemary EO respectively, showed two populations of nanoparticles, having small (30 nm) and large (235 nm) mean size, while NLC CEO3 (rosemary EO 3%) contained a single population of small nanoparticles (Figure 2).

These results point out that the ratio solid lipid/liquid lipid plays a key role in determining NLC size and dimensional distribution, as already reported in literature [31]. The interactions occurring among NLC components may change depending on the solid lipid/liquid lipid ratio. These interactions may affect the nanoparticle curvature radius, thus leading to nanoparticles with different mean sizes.

**Figure 3:**
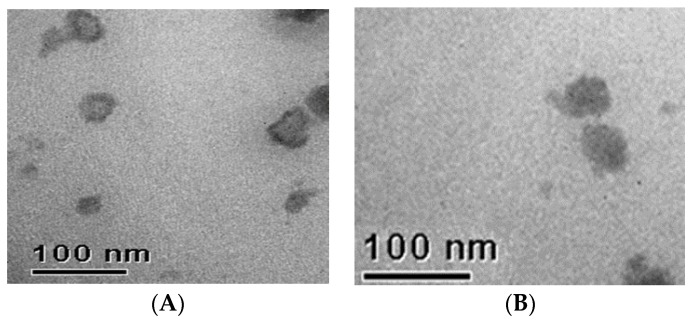
Transmission electron microscopy (TEM) images of solid lipid nanoparticles A (**A**); and lipid nanoparticles CEO3 (**B**).

Morphological analyses were performed by transmission electron microscopy (TEM) only on samples showing a single population of nanoparticles. TEM imaging of SLN A and NLC CEO3 showed that nanoparticles were round in shape (Figure 3). No evident difference could be observed between SLN A and NLC CEO3 TEM images as TEM provides information only about the morphology and the external surface of the nanoparticles.

While the content of rosemary EO strongly affected the mean particle size and dimensional distribution, zeta potential values were similar for all the nanoparticles under investigation. It is interesting to note that all rosemary EO-loaded nanoparticles showed zeta potential similar to that of unloaded SLNs, thus suggesting that the incorporation of rosemary EO into these nanoparticles did not influence their superficial charge.

**Figure 4:**
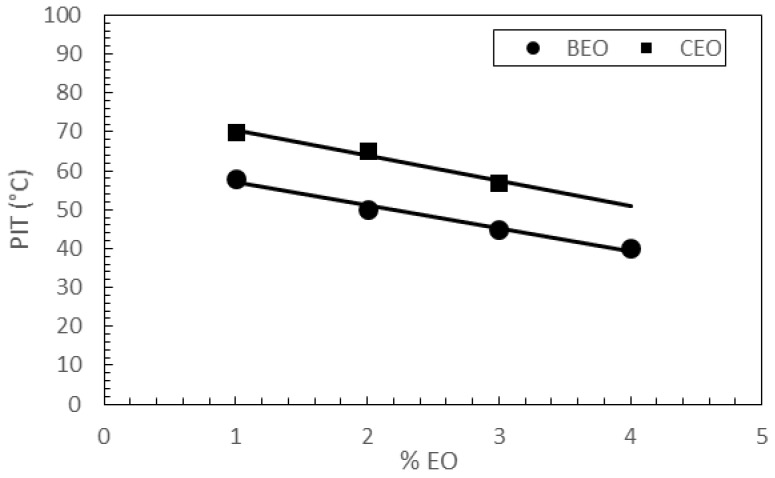
Relationship between the percentages of essential oil (EO) in NLC BEO1–4 and CEO1–3, and the corresponding phase inversion temperature (PIT) values determined during their preparation.

The data reported in Table 3 highlight that the higher the rosemary EO content, the lower the PIT values, for both series of NLCs. As shown in Figure 4, a linear relationship between percentages of rosemary EO used to prepare the nanocarriers and PIT values of the corresponding nanoparticles was observed (*r*^2^ = 0.985 for NLC BEO1–4; *r*^2^ = 0.983 for NLC CEO1–3).

Concentration-dependent interactions occurring between rosemary EO and lipid nanoparticles components could explain PIT lowering as rosemary EO content increased. As reported in the literature [32], rosemary EO hydrophilic lipophilic balance (HLB) is 16.5 while cetyl palmitate has an HLB value of 10.0. Therefore, increasing the percentage of rosemary EO in the lipid phase would lead to an increase in hydrophilicity of the lipid mixture. In conventional emulsions, an increase in hydrophilicity is expected to lead to an increase in PIT values [33]. As NLC BEO1–4 and CEO1–3 contain both solid and liquid lipids, they could be classified as multiple NLCs [17]. Being comprised of complex structures, with a matrix consisting of oil droplets embedded in solid lipids and a shell of surfactants, the interactions among different components could affect PIT values differently from conventional emulsions. Therefore, further studies have been planned to elucidate the interactions occurring between rosemary EO and other nanoparticle constituents.

As only NLC CEO3 showed a single population of nanoparticles, we selected this colloidal dispersion for stability tests. No significant changes of particles size and polydispersity indexes were observed during storage at room temperature for two months (data not shown). Therefore, we used NLC CEO3 to prepare the gel vehicles for in vivo topical application.

All gels showed no change in their organoleptic properties during storage in non-transparent containers for one month at room temperature.

### 3.2. In Vivo Evaluation of Gel Formulations

The ability of SLNs and NLCs to increase drug skin permeation has been attributed to different factors such as occlusive properties, specific drug-carrier interactions with the skin and close contact with the skin’s outermost layers due to their small size [34,35,36]. After topical application, SLNs and NLCs form a continuous and dense film on the cutaneous surface that prevents or decreases water loss from the skin, thus increasing skin hydration and, hence, drug skin permeation [37]. Wissing and Müller [38] performed an investigation on human volunteers, studying the hydrating and viscoelasticity effects of an O/W cream containing SLNs in comparison with the same vehicle without SLNs. The results of this study showed that the cream containing SLNs provided a higher increase in skin hydration than the conventional formulation, while skin elasticity was not significantly affected after a 28-day treatment with these formulations, likely owing to the young age of the volunteers enrolled in the study.

Therefore, in this work, the effects of rosemary EO-loaded lipid nanoparticles on skin hydration and elasticity from gel vehicles was assessed using a gel containing unloaded SLNs as a control.

We chose gel formulations as a vehicle for rosemary EO-loaded NLCs in order to avoid the interactions between vehicle components and NLCs, which may occur in complex vehicles such as emulsions and could interfere with the ability of NLCs to affect skin hydration and elasticity.

**Figure 5:**
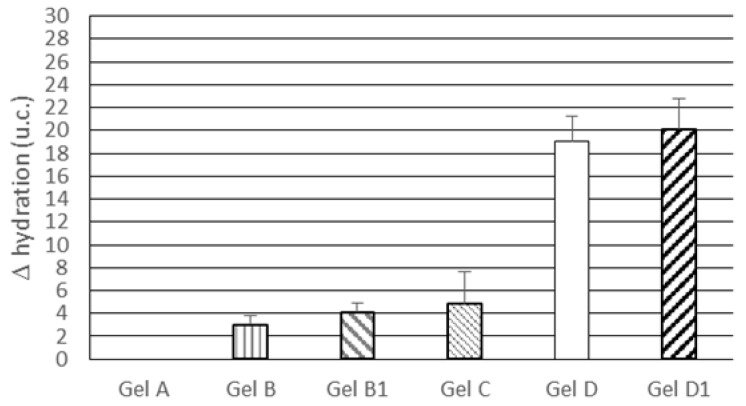
Skin hydration difference (Δ hydration) between baseline values and values recorded after a one-week treatment with the gels under investigation. Statistically significant differences (*p* < 0.05) were observed for the following comparisons: all gels vs. gel A; gel D vs. gels B, B1 and C; gel D1 vs. gels B, B1 and C.

The results of in vivo tests on skin hydration were expressed as hydration difference (Δ hydration) between baseline values and values recorded after a one-week treatment with the gels under investigation. As shown in Figure 5, topical treatment with gel A, which did not contain the active ingredient or lipid nanoparticles, did not lead to any change in skin hydration. Gels B and B1, incorporating 1.5% and 3.0% *w*/*w* free rosemary EO, respectively, enhanced skin hydration but no significant difference (*p* > 0.05) was observed between Gel B and B1, thus suggesting that an increase in rosemary EO concentration in the vehicle was not effective in further improving skin hydration. Topical application of gel C, containing only unloaded SLN, resulted in a hydration improvement similar to that obtained from gel B and B1, thus confirming the ability of SLNs to act as a skin-hydrating factor. After a one-week treatment, rosemary EO-loaded NLCs in gel vehicles (gels D and D1) provided Δ hydration values greater than those observed for gels B, B1 and C, but no significant difference (*p* > 0.05) was observed between gels containing different percentages of rosemary EO-loaded NLC. These results highlight the usefulness of loading rosemary EO into lipid nanoparticles, supporting the findings of previous studies on rosemary extracts that reported an increase in activity upon loading these extracts into nanoparticles [39,40].

**Figure 6:**
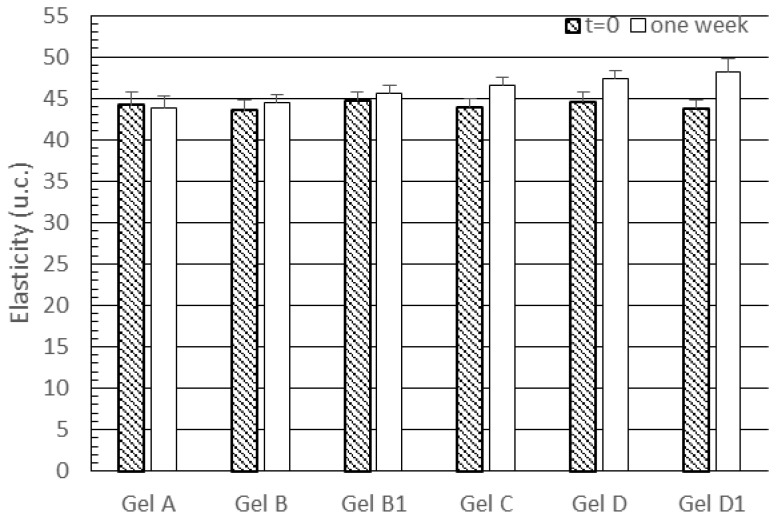
Skin elasticity values recorded before treatment (*t* = 0) and after a one-week treatment with the gels under investigation. Statistically significant differences (*p* < 0.05) were observed after one week for the following comparisons: gel C vs. gels A, B, B1, D and D1; gel D vs. gels A, B, B1 and C; gel D1 vs. gels A, B, B1 and C.

Figure 6 shows that skin treatment with gels A, B and B1 did not alter elasticity values, while all the gels containing lipid nanoparticles provided a slight but significant increase in skin elasticity (*p* < 0.05 for all comparisons between gels containing lipid nanoparticles and gels without nanoparticles). However, it is interesting to note the all subjects showed high elasticity values before the treatment with the gels under investigation. Therefore, only moderate increases of this parameter could be expected. Analogously to skin hydration data, no significant difference was observed between elasticity values obtained from gels D and D1 (*p* < 0.05). In addition, our data showed a moderate effect on skin elasticity of the gel containing unloaded SLNs (gel C), which contrasts with the results reported by Wissing and Müller [38], who did not find any change in this parameter upon topical application of SLNs. The effect of unloaded SLNs on skin elasticity observed in our work could be attributed to the older age of volunteers enrolled in this investigation. However, gel C provided a significantly lower increase in skin elasticity than gels D and D1 (*p* < 0.05 for both comparisons), thus proving better activity from lipid nanoparticles loading rosemary essential oil.

In conclusion, the results of this study pointed out that in vivo topical application of rosemary essential oil from gel vehicles showed a hydrating effect that remarkably increased upon rosemary EO loading into lipid nanoparticles. A slight but significant effect on skin elasticity could be detected only after topical application of gel vehicles containing unloaded SLNs or rosemary EO-loaded NLCs. Therefore, loading rosemary essential oil into lipid nanoparticles seems to be a promising strategy to improve its topical benefits and for designing topical formulations for the treatment of cutaneous alterations involving loss of skin hydration and elasticity.

## Figures and Tables

**Table 1:** Composition (% *w*/*w*) of the lipophilic phase of unloaded and rosemary essential oil (EO)-loaded lipid nanoparticles. Ingredients include Oleth-20, Glyceryl Oleate (GO), Cetyl Palmitate (CP) and essential oils.

Code	Ingredients			
	Oleth-20	GO	CP	EO
A	9.0	5.0	7.0	--
BEO1	9.0	5.0	6.0	1.0
BEO2	9.0	5.0	5.0	2.0
BEO3	9.0	5.0	4.0	3.0
BEO4	9.0	5.0	3.0	4.0
CEO1	9.0	5.0	7.0	1.0
CEO2	9.0	5.0	7.0	2.0
CEO3	9.0	5.0	7.0	3.0

**Table 2:** Gel composition (% *w*/*w*). Water contained 0.35% *w*/*w* imidazolidinyl urea and 0.05% *w*/*w* methylchloroisothiazolinone and methylisothiazolinone as preservatives. Ingredients include Carbopol, Triethanolamine (TEA), essentials oils, Solubilisant, solid lipid nanoparticles (SLNs) preparation A, lipid nanoparticles (NLCs) preparation CEO3 and water.

Code	Ingredient						
	Carbopol	TEA	EO	Solubilisant	SLN A	NLC CEO3	Water
A	0.8	0.8	--	--	--	--	98.4
B	0.8	0.8	1.5	4.5	--	--	92.4
B1	0.8	0.8	3.0	9.0	--	--	86.4
C	0.8	0.8	--	--	98.4	--	--
D	0.8	0.8	--	--	--	49.2	49.2
D1	0.8	0.8	--	--	--	98.4	--

**Table 3:** Mean size (Z-Ave ± standard deviation (S.D.)), polydispersity index (PDI ± S.D.), ζ potential (± S.D.) and phase inversion temperature (PIT) of unloaded and rosemary oil-loaded lipid nanoparticles.

Code	Z-Ave (nm)	PDI	ζ potential ± S.D. (mV)	PIT (°C)
A	36.5 ± 1.45	0.208 ± 0.010	−1.55 ± 0.27	72
BEO1	26.9 ± 1.23	0.341 ± 0.021	−1.76 ± 0.38	58
BEO2	27.6 ± 1.01	0.368 ± 0.018	−2.05 ± 0.37	50
BEO3	141.9 ± 5.43	0.495 ± 0.022	−1.99 ± 0.39	45
BEO4	171.7 ± 4.66	0.336 ± 0.019	−1.78 ± 0.45	40
CEO1	34.8 ± 1.10	0.367 ± 0.017	−2.19 ± 0.44	70
CEO2	31.6 ± 1.92	0.325 ± 0.020	−1.89 ± 0.58	65
CEO3	28.1 ± 1.02	0.171 ± 0.011	−1.93 ± 0.66	57
